# Biopreservative Efficacy of Bacteriocin GP1 of *Lactobacillus rhamnosus* GP1 on Stored Fish Filets

**DOI:** 10.3389/fnut.2019.00029

**Published:** 2019-03-22

**Authors:** A. R. Sarika, Aaron P. Lipton, M. S. Aishwarya

**Affiliations:** ^1^Kerala State Council for Science, Technology and Environment, Thiruvananthapuram, India; ^2^Centre for Marine Science and Technology, Manonmaniam Sundaranar University, Kanyakumari, India

**Keywords:** *Lactobacillus rhamnosus* GP1, bacteriocin GP1, Nisin B440, sodium benzoate, biopreservative, fish filets

## Abstract

The bacteriocin based strategy of biopreservation has got wide spread research interests in the recent past for their prospects in reducing usage of chemical preservatives. The bacteriocin GP1 with antibacterial activity and produced by *Lactobacillus rhamnosus (L. rhamnosus)* GP1 was tested for its effect on sensory (color, odor, and appearance), chemical (pH, Total Volatile Base-Nitrogen (TVB-N), Total Methyl Amine (TMA), Total Free Fatty Acid) and bacteriological (total bacterial count, count of *Staphylococcus* sp., *Aeromonas* sp., total coliform, *Lactobacillus* sp., *Pseudomonas* sp., and *Vibrio* sp.) quality attributes of fish filets stored at 4 and 0°C. The sensory attributes of the fish filets treated with the bacteriocin and control from 7 to 28 days of storage in both the storage temperatures varied significantly. The pH of the raw fish increased from the initial 6.8 to 7.91 and 7.43 for the control and bacteriocin GP1, respectively, at the end of storage period (28 days) when stored at 4°C. However, the pH showed a decreasing trend with the increase in period of storage for the samples stored at 0°C. The TVB-N content of the bacteriocin treated samples stored at 4°C remained within the limit of acceptability (35 mg/100 g) at the 21st day. The TMA level also remained within the acceptable limit of 10–15 mg/100 g at the 21st day in the case of bacteriocin-treated samples. The application of bacteriocin GP1 in the stored fish was effective in controlling the growth of coliforms, *Aeromonas* sp., *Lactobacillus* sp., and *Vibrio* sp. in the treated fish samples. The study concluded the prospects of bacteriocin GP1 as a biopreservative in storage of fish and fish products.

## Introduction

The seafood industry is constantly searching new technologies for storage of fresh fish as they are highly perishable due to their biological composition. Chemical preservatives and other conventional preservation strategies fail to deliver the requisite health benefits and cause serious disorder thus necessitates seeking alternatives. The major cause for the spoilage of fresh and preserved fish is the microbial growth and metabolic activity (1). Recent research on to the same centers around the biopreservative strategy which consists of inoculating food product by selected bacteria and its antibacterial products which deter the growth of spoilage causing micro-organisms, without altering the product quality (2).

Lactic acid bacteria (LAB) have huge prospects for use in biopreservation. The GRAS status together with the conviction of being safe to consume permits their usage in foods without additional regulatory approval. Bacteriocins of LAB with potent antibacterial activity against food spoilage and pathogenic bacteria have been used as a natural food ingredient, essentially as a biopreservative. The bacteriocins have been consumed for millennia by mankind as products from LAB which relates the fact that the only commercialized food grade bacteriocins available till date are purified from them. Further, being peptides, bacteriocins are assumed to be easily degraded by the proteases in the gastrointestinal tract and hence are considered as good biopreservatives. Most of the studied bacteriocins possess good thermo stability thus able to remain stable during thermal processing cycle of foods, while the others can act at low pH and temperature thus finds application in acid foods and cold-stored products (3). Many bacteriocins have been isolated for use as natural food biopreservative (4, 5). Nisin produced by *Lactococcus lactis* sub sp. *lactis*, is a bacteriocin approved for application in the food industry for its activity against Gram positive foodborne pathogenic microorganisms (6). Previous studies have shown the effective application of nisin as a biopreservative in processed fish (7–9).

The inhibitory potential of the bacteriocin GP1 produced by the LAB, *Lactobacillus rhamnosus (L. rhamnosus)* GP1 has been demonstrated against a number of spoilage-producing and pathogenic bacteria (10). This study was aimed to find out the effect of application of the bacteriocin GP1 on microbiological, chemical, and sensory quality of high value fish filets stored at 0 and 4°C in comparison with the chemical preservative sodium benzoate and the bacteriocin nisin B440.

## Materials and Methods

### Growth and Culture of Bacteria

The bacteriocin producing LAB strain *(L. rhamnosus)* GP1 (10) was grown in MRS medium (11) and incubated at 30°C. *L. brevis* BF1 was used as the indicator strain to assay the bacteriocin. Both the strains were stored in MRS agar (Hi-Media) slants and propagated as and when required.

### Bacteriocin Preparation and Determination of Activity

Five milliliters of working culture of *L. rhamnosus* GP1 was transferred to 1,000 ml of modified MRS broth (supplemented with 0.1%; w/v of yeast extract, 0.1%; v/v of tween 80, and 2.0%; w/v of glucose) to maximize the production of bacteriocin. The culture broth was centrifuged after growth (72 h at 30°C; pH 3.75) at 10,000 rpm for 30 min and the cell free culture supernatant was collected. The supernatant fluid was adjusted to pH 7.0 with 0.1 N NaOH and was filter sterilized (0.45 μm pore size) under vacuum. The bacteriocin GP1 was partially purified by pH-adsorption (12) and activity determined. For detecting the bacteriocin activity, 10 μl of the cell free filtrate was placed on an agar plate containing an overlay of indicator cells. The inhibitory activity against the indicator organisms was observed after incubating the agar plate for 24 h. Antimicrobial activity was expressed as arbitrary units (AU). Each arbitrary unit is the reciprocal of highest dilution showing a clear zone of growth inhibition (13).

### Fish Preparation and Sampling

The fish used in the study belonged to Grouper (Reef Cod) family and was obtained from Vizhinjam Fish Landing Center, Thiruvananthapuram, Kerala, India. The fish weighing 11.8 kg was immediately brought to the laboratory in insulated container and washed in potable water. The fish as a whole was dipped in chlorine water, washed with sterile water, beheaded and eviscerated. Then it was allowed to bleed for 15 min and washed again in sterile water and skin removed, deboned, and fileted (10 g each) using a sterile sharp knife. The filets were surface sterilized by exposure to UV light at 265 nm (Arklite, India) for 15 min and sterility determined by microbiological examination. Four different preservative solutions were prepared with 800 AU/ml nisin (produced by *Lactococcus lactis* MTCCB440), 1,200 AU/ml bacteriocin GP1, 0.1% of sodium benzoate and autoclaved distilled water which served as the control. The prepared preservative solutions (2 ml each) were sprayed evenly over the fish samples (10 g) with a sterile hand-held sprayer and kept intact for 20 min. The treated fish pieces were enfolded in sterile aluminum foil, placed in separate sterile boxes, and stored at temperatures *viz*., 4 and 0°C. At 0 (within 20 min of treatment), 7, 14, 21, and 28 days of refrigeration, samples from each treatment were taken out subjected to different analyses—microbiological, physicochemical, and sensory. All the analyses were done in triplicate.

### Analysis of Bacterial Count

For bacteriological analysis, 1 g slice of the stored filet was made using sterile blade and homogenized for 2 min using sterile mortar and pestle. The samples were serially diluted and plated in triplicate on nutrient agar and selective agar plates such as Eosin Methylene Blue (Hi-Media), Thiosulfate Citrate Bile Salts Sucrose (TCBS) agar (Hi Media), de Mann Rogosa Sharpe (MRS; Hi Media) agar, and Pseudomonas Selective Agar (Hi Media) at 30°C for 48 h.

### Sensory Evaluation

The odor and appearance of fish samples were analyzed for their sensory characteristics using a 9-point hedonic scale (14); 1 being dislike extremely and 9 like extremely (15) using five trained panelists.

### pH Measurements

The samples stored at different temperatures were subjected to determination of change in pH. The samples (1 g each) was homogenized using 9 ml distilled water and pH determined using a Cyberscan model 500 pH meter (Euteon Instruments, Singapore). The results were expressed as the mean of the determinations.

### Total Volatile Basic Nitrogen (TVB-N) and Total Methyl Amine (TMA) Measurements

Conway's method was employed for determination of TVB-N and TMA of the samples. For analyses, the 2 g of sample from each experimental set and homogenized with 10 ml of 4% trichloroacetic acid which was filtered through a Whatman No.1 filter paper. The filtrate was used for analyses of TVB-N and TMA. The experiments were carried out following the procedure of Conway (16).

### Statistical Analysis

The experiments were done in triplicate. Data were analyzed using statistical method ANOVA on 5% significance level in Excel 2010.

## Results

### Bacteriocin Preparation

The LAB strain *(L. rhamnosus)* GP1 (10) was used to extract the bacteriocin GP1 to study its application as biopreservative in chill-stored high value marine fish. The growth of the strain in modified MRS broth showed a drastic increase between 24 and 60 h after inoculation (log phase). A gradual stationary and a steady decline phase were obtained subsequent to this slope. The maximum production of bacteriocin GP1 was noted in the stationary phase, at 72 h. The partially purified bacteriocin GP1 was collected following centrifugation of the culture and pH-adsorption of the cell free culture supernatant.

### Microbiological Analyses

**Figure 1:**
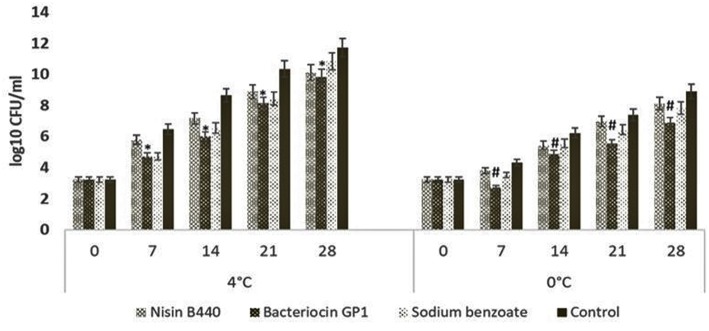
Change in total viable count of the fish samples stored at 4 and 0°C. The bar graphs show mean ± SD. ^*^indicates a significant difference between bacteriocin GP1 treated samples and control (*P* = 0.02) stored at 4°C, ^#^indicates a significant difference between bacteriocin GP1 treated samples and control (*P* = 0.019) stored at 0°C.

The total bacterial count of the fish sample stored at 4 and 0°C are presented in Figure 1. The microbial load increased with increase in the storage period at 4°C irrespective of the treatments employed. The initial mean bacterial load was 3.24 ± 0.11 CFU/g in all the experimental groups. Compared with the control which started deteriorating after 7 days of storage, the growth of spoilage bacteria was significantly inhibited in treated samples; the treatment with bacteriocin GP1 and sodium benzoate managed to extent the fish shelf life up to the 14th day of storage.

**Figure 2:**
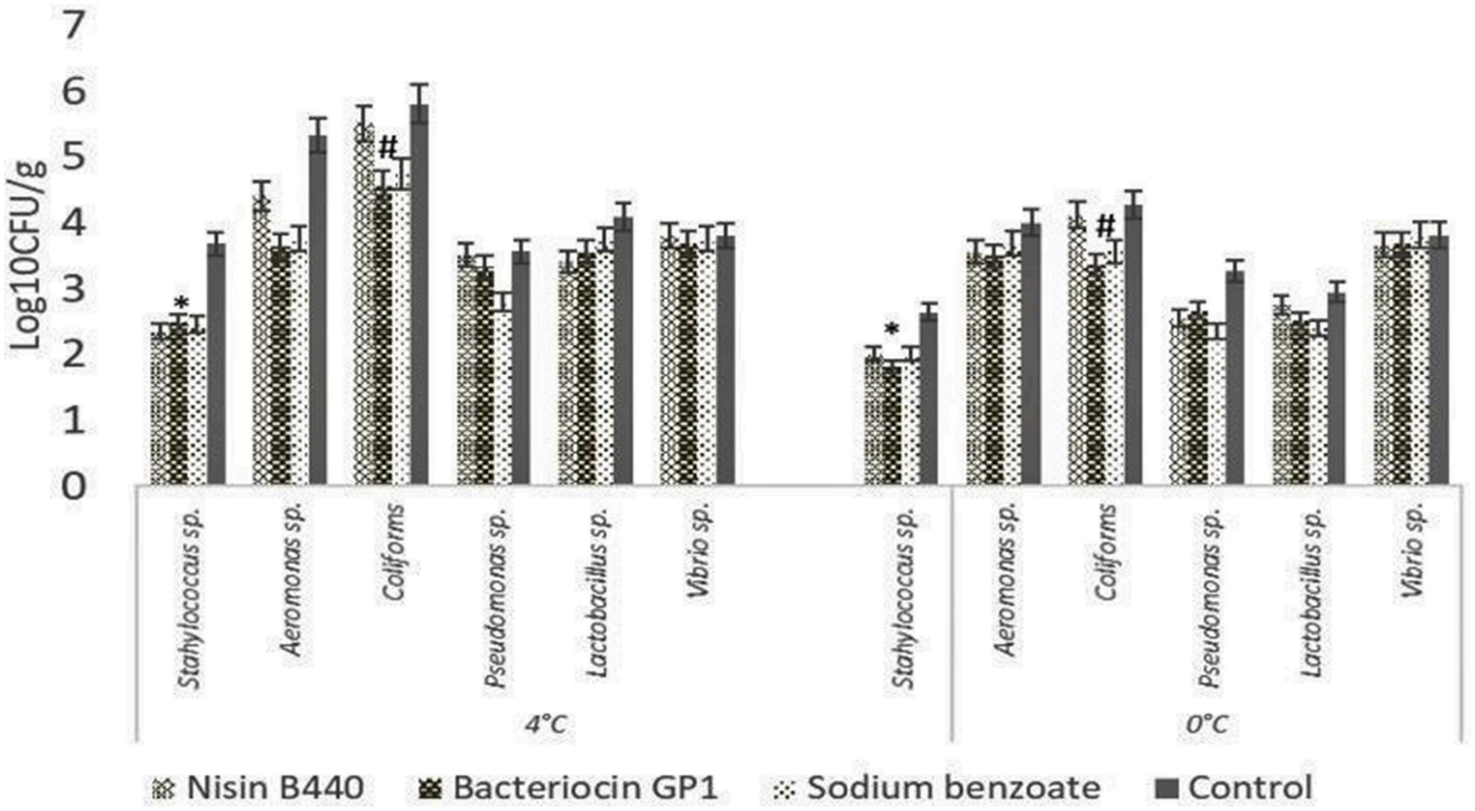
Change in specific bacterial count of stored fish filets on the 7th day of storage. The bar graphs show mean ± SD. ^*^indicates a significant difference in *Staphylococcus* count between different treatments (*P* = 0.045) stored at 4 and 0°C, ^#^indicates a significant difference in Coliform count between different treatments (*P* = 0.008) stored at 4 and 0°C.

The results of specific bacterial counts, obtained from samples at the 7th day of storage, are shown in Figure 2. The bacteriocin GP1, nisin B440, and the chemical preservative sodium benzoate significantly inhibited the psychrophiles compared to the control. The psychrophilic bacterial count increased with increase in storage period up to 28 days (data not shown). The load of *Staphylococcus* sp., *Aeromonas* sp., coliforms, *Lactobacillus* sp., *Pseudomonas* sp., and *Vibrio* sp. followed an increasing trend initially up to the 14th day, which became stable till 28th day. While a reduction in the spoilage bacterial count was noted for all the treated samples compared to the control, bacteriocin GP1 was effective in controlling the growth of coliforms, *Aeromonas* sp., *Lactobacillus* sp., and *Vibrio* sp.

### Sensory Evaluation

**Figure 3:**
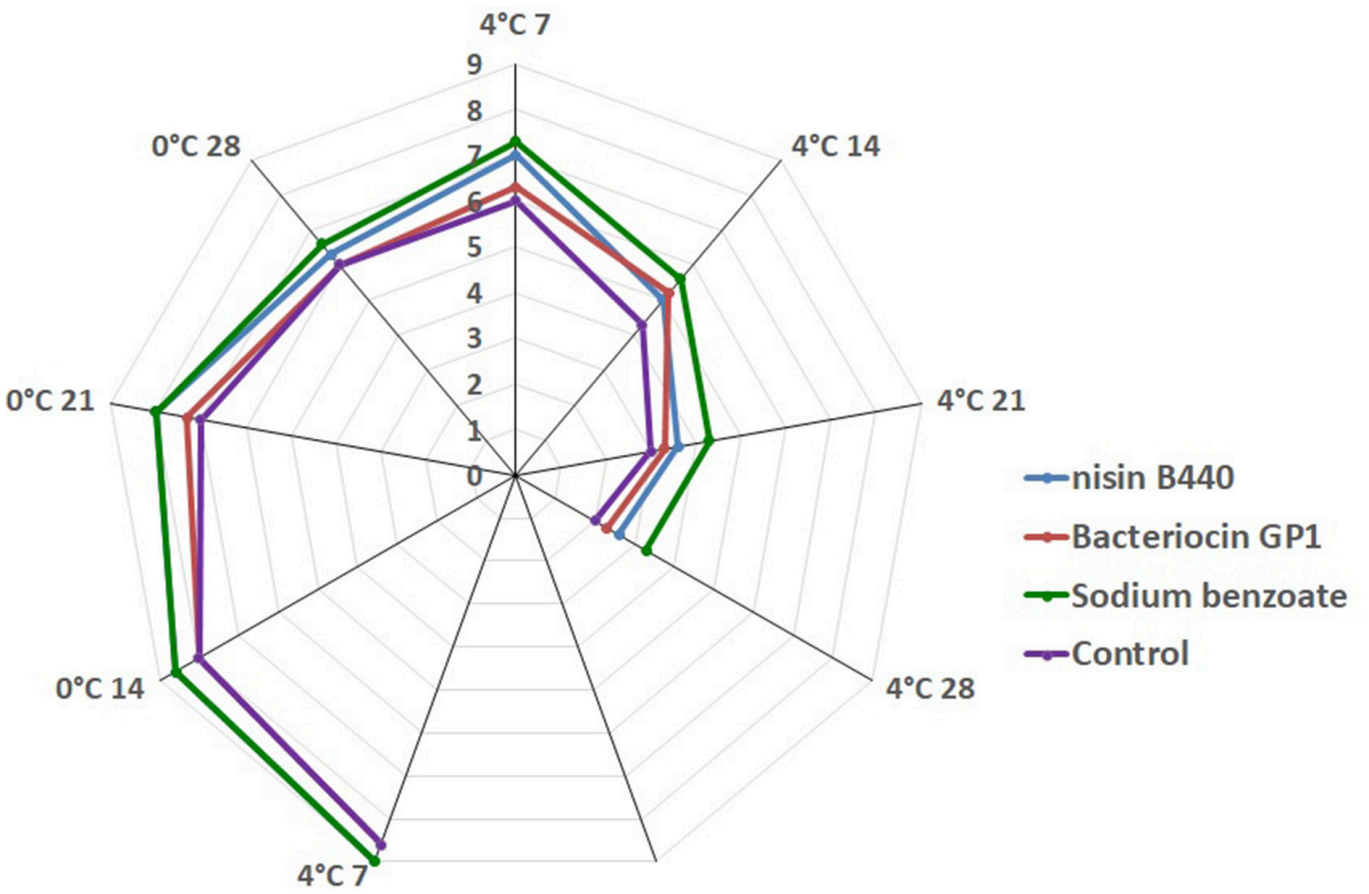
Hedonic Score on four different treatments (Sample in day 0 were not included as they were judged excellent).

The sensory scores decline with storage (Figure 3). Though, the level of acceptability of the fish sample decreases as the storage time increases in both the treated and untreated fish samples, significant difference (*P* < 0.05) was noted in the sensory attributes in case of the treated fish samples compared to the control from 7 to 28 days of storage. The fish sample treated with nisin B440, bacteriocin GP1, and sodium benzoate had 5.0 ± 0.0, 5.2 ± 0.4, and 5.6 ± 0.4 score, respectively at day 14 of storage at 4°C while the control had 4.3 ± 0.4 score at the same time of storage. The overall scores were beyond the acceptable limit (4.0) at the end of the storage period (28 days) at 4°C.

### Change in pH

The pH of the fish samples showed an increasing trend with increase in storage period. Table 1 summarizes the average values analyzed on each sampling day. For the fish samples stored at 4°C, the pH values showed an increased trend in both the control and experimental sets. The range in pH was from 6.8 ± 0.01 to 7.91 ± 0.01 as determined from the initial day to the end of storage period (28th day) in case of control as against the treated samples which maintained the pH range of <7.0 till the 21st day of storage. Significant differences (*P* < 0.05) were noted in the pH of the fish samples treated with the bacteriocin and control from 7 to 28 days of storage in both the storage temperatures.

**Table 1:** pH changes of the fish filets treated with bacteriocin preparations stored at 4 and 0°C for 28 days; the values are shown as show mean ± SD.

**Treatments**	**4°C**					0°C^*^				
	**0**	**7**	**14**	**21**	**28**	**0**	**7**	**14**	**21**	**28**
Nisin B440	6.8 ± 0.01	6.84 ± 0.01	6.90 ± 0.00	6.99 ± 0.02	7.50 ± 0.01	6.82 ± 0.01	6.76 ± 0.01	6.75 ± 0.01	6.69 ± 0.01	6.62 ± 0.01
Bacteriocin GP1	6.8 ± 0.01	6.82 ± 0.01	6.85 ± 0.01	6.94 ± 0.01	7.43 ± 0.02	6.8 ± 0.01	6.75 ± 0.01	6.73 ± 0.00	6.67 ± 0.01	6.62 ± 0.02
Sodium benzoate	6.8 ± 0.01	6.84 ± 0.01	6.87 ± 0.01	6.98 ± 0.01	7.41 ± 0.01	6.81 ± 0.01	6.76 ± 0.01	6.72 ± 0.01	6.66 ± 0.01	6.60 ± 0.01
Control	6.8 ± 0.01	6.87 ± 0.02	7.01 ± 0.01	7.25 ± 0.01	7.91 ± 0.01	6.82 ± 0.01	6.79 ± 0.02	6.77 ± 0.01	6.70 ± 0.01	6.65 ± 0.01

* *Indicates a significant difference in pH between bacteriocin GP1 treated samples and control (P = 0.001) stored at 0°C*.

### Changes in TVB-N

**Figure 4:**
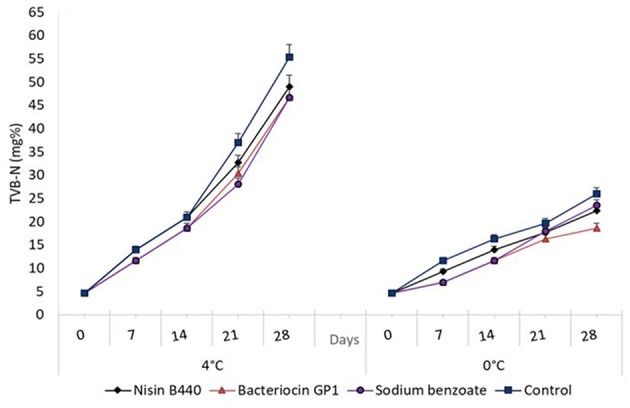
Changes in the total volatile base nitrogen (TVB-N) content (mg%) in fish filets stored at 4 and 0°C; values represent means ± SE, Significant difference is defined at *P* < 0.05.

The TVB-N values of the treated samples and control are shown in Figure 4. The average TVB-N content in the fish samples was 4.67 ± 4.0 and 55.33 ± 4.0 mg of nitrogen / 100 g of fish flesh, respectively, at 0 and 28 days stored in 4°C. By day 21, the TVB-N value of control had increased from 21 to 37 mg/100 g of fish. For the bacteriocin treated samples, although it increased with storage; from day 7, it showed a reduced level when compared to the control. For, the samples stored for 21 days, the TVB-N content showed a significant difference (*P* < 0.05) which compared with the control, the values were 32.67 ± 4.0, 30.33 ± 4.0, and 28.0 ± 7.0 mg of Nitrogen/100 g for nisin B440, Bacteriocin GP1 and sodium benzoate treated samples, respectively, as against 37.0 ± 4.0 mg of Nitrogen/100 g observed for the untreated (control) samples. The pattern of TVB-N production in 0°C was consistent with that observed for 4°C in all experiments with significant difference noted (*P* < 0.05) for treated samples vs. control (Figure 4), though remained within the limit of acceptability (30–35 mg/100 g) even at the end of storage period.

### Changes in TMA

**Figure 5:**
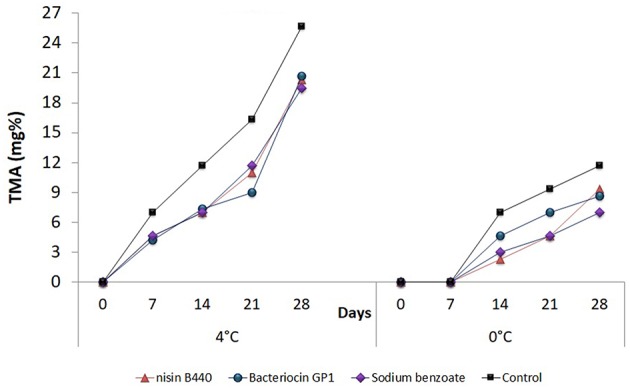
Changes in the total methyl amine (TMA) content (mg%) in fish filets stored at 4 and 0°C; values represent means ± SE, Significant difference is defined at *P* < 0.05.

The measurements made in terms of TMA are presented in Figure 5. The TMA content also followed an analogous trend to that of TVB-N when stored at 4°C where a significant increase was observed (*P* < 0.05) with storage period. Initial TMA content was 0 for both the untreated and treated samples. The values in the 28th day were 20.33 ± 4.0, 20.67 ± 4.0, and 19.5 ± 0.0 for Nisin B440, Bacteriocin GP1and sodium benzoate treated samples, respectively, as against 25.67 ± 4.0 observed in case on the untreated (control) sample. It could also be observed that the TMA values for the samples stored at 0°C were lower than the samples stored at 4°C though significant differences amongst the treatments were noticed as is evidenced statistically. However, the values remained within the range of acceptability (10–15 mg/100 g) even after 28 days of storage at 0°C in both the treated samples and the control.

## Discussion

Biopreservation approaches for food items are gaining importance and interest among the industry and the consumers. The biopreservative efficiency of several bacteriocins had been proved earlier (17–19) in different food systems *viz*., fish, meat, and vegetable products. The effectiveness of bacteriocin based biopreservatives had been extensively tested in meat products (19, 20) and in vegetables (21). However, scanty information is known regarding preservation of seafood using bacteriocins. The present study targeted on investigating the preservative efficacy of the studied bacteriocin GP1 produced by *(L. rhamnosus)* GP1 in a high value fish sample stored under different temperatures (4° and 0°C).

There was an increase in total bacterial count during storage at 4°C irrespective of the treatments employed. The results of increased viable count above 8 log_10_ CFU/g in all samples after 14 days of storage period were similar to earlier findings (22). The bacterial count could have increased due to the ambient congenial growth conditions at the higher temperatures of storage (23). To the observations of Randazzo et al. (18), the survival and persistence of natural contaminant organisms in the fish is the result of high final load detected throughout the period of storage. Up on comparing the bacterial load among the different treatments, it was evidenced that the bacteriocin treated samples slowed down the bacterial proliferation when compared with the untreated samples, the efficacy of bacteriocin GP1 in controlling the spoilage bacteria is in par with the biopreservative nisin and chemical preservative sodium benzoate. Since the load of bacteria in GP1 treated samples (5.99 ± 0.04 log_10_ CFU/g) remained within the acceptability limit at the 14th day, it could be concluded that this bacteriocin could extent the shelf-life of the fish. The potential of the bacteriocins to control the spoilage bacteria in fish filets are in consistence with the observations made with Pediocin 31-1 (19) in pork meat.

The proliferation of the psychrophilic bacteria (24) in the fish filets occurred with increase in storage period at the higher temperature of 4°C. Liu et al. (25) isolated psychrophiles from tray-packed Tilapia filets stored at 0°C. Similar to this observation, the cod fish flesh stored at 0°C harbored a definite load of the psychrophiles, which followed a stable trend throughout the period of storage in the case of the untreated sample. However, the observations with the bacteriocin treated samples were encouraging in that it could limit the growth of at least a few strains of bacteria.

The sensory characteristics of fish are clearly noticeable to the consumer and are extremely important for consumer satisfaction (26). The observations made for odor and appearance were considered for determining the sensory attributes of the stored fish sample. The fish samples at the initial day i.e., just before storage had the best sensory attributes and scored 9.0. However, the acceptability scores for odor and appearance of the fish stored at 4 and 0°C decreased with the progression of storage. The fish scored “liked slightly” during the first 7 days of storage and scored “dislike slightly” between 7 and 14 days of storage for control group samples (Figure 3). The fish samples reached the acceptability quality limit of 4.0 as per the protocol of Renitta et al. (27) at the 14th day for the control samples and after 15 days for the bacteriocin treated samples. The fish sample treated with bacteriocin GP1 had 5.2 ± 0.2 score at day 14 at 4°C while the control had 4.3 ± 0.4 score at the same time of storage. There were significant differences in the sensory attributes for the fish samples treated with the bacteriocins and control at the 21st and 28th day of storage, though the samples have become unacceptable.

The pH changes in the fish flesh is determinant of the decomposition of nitrogenous compounds during storage thus producing alkaline compounds. This high post-mortem pH (28, 29) indicates the bacterial growth, loss of quality and possible spoilage. The pH of the raw fish used in the study was in the range of 6.8 in the beginning, which changed drastically upon storage at 4°C. Contrary to observation made at 4°C, the pH showed a decreasing trend with the increase in period of storage for the samples stored at 0°C.

Total volatile bases nitrogen (TVB-N) measurements quantify the contents of trimethylamine (TMA), dimethylamine (DMA), ammonia, and other volatile basic nitrogenous compounds associated with seafood spoilage (30, 31). A significant increase of TVB-N values (10.48 ± 0.07 mg/100 g) was recorded in the stored fish (*P* < 0.05). The TVB-N content of the untreated (control) fish sample stored at 4°C exceeded the maximum level for acceptability for marine fish i.e., 35 mg/100 g (32), after 21 days. This value agrees with TVB-N levels of sea bass (33) and Malawi Tilapia (34) stored in ice. In the case of the bacteriocin treated samples, it remained within the limit of acceptability at the 21st day of storage. The observations in tune with this had been made earlier in Pediocin 31-1 in which the 80 AU/ml of this bacteriocin maintained TVB-N within the acceptability limit to 15 days as against the control (19).

The content of TMA was below detectable level (0) in the case of cod fish when determined initially. Earlier observation (35) showed TMA content to range from of 0.8 to 4 mg/100 g in fresh marine fishes. TMA values increased throughout the time of storage in the present study; the level of 10–15 mg/100 g being the maximum limit of acceptability (36) to indicate fish freshness. This level was attained in the untreated (control) fish sample at the 14th day, whereas in the bacteriocin treated sample remained within the limit of acceptability even at the 21st day of storage and thereafter became unacceptable. Thus, it was noted that the bacteriocin GP1 was most effective in restricting the production TVB-N and TMA, thereby increasing the shelf-life of the fish at 4°C.

The reduction in the load of the bacteria and all other parameters observed with the bacteriocin treated samples were analogous with the experiments conducted with the chemical preservative sodium benzoate as is evidenced from the results of this study. The preservation of the fish at the ambient (25 ± 2°C) or lower temperatures (4°C) requires the use of chemical preservatives viz., benzoates, nitrites, sulphites, and sorbates (37) or tedious processing (38) steps to enable the shelf-life extension. But as the chemical preservatives are associated with undesirable side effects (39), a replacement is required which does not influence the organoleptic attributes of the product. The findings made in this study could be considered noteworthy as there is a likelihood that bacteriocin GP1 could be further purified and used as preservative for storing high value marine fish.

## Data Availability

The datasets generated for this study are available on request to the corresponding author.

## Author Contributions

All authors listed have made a substantial, direct and intellectual contribution to the work, and approved it for publication.

### Conflict of Interest Statement

The authors declare that the research was conducted in the absence of any commercial or financial relationships that could be construed as a potential conflict of interest.
